# Development and Characterization of Chitosan–PVA–Tannic Acid Film for Extended Shelf Life and Safety of Food Products

**DOI:** 10.1021/acsomega.4c09964

**Published:** 2025-05-05

**Authors:** Sakshi Jasrotia, Sonali Gupta, Manas Laxman Kudipady, Yashoda Malgar Puttaiahgowda

**Affiliations:** † Department of Chemistry, Manipal Institute of Technology, 76793 Manipal Academy of Higher Education, Manipal, Karnataka 576104, India; ‡ Department of Information and Communication Technology, Manipal Institute of Technology, Manipal Academy of Higher Education, Manipal, Karnataka 576104, India

## Abstract

The growing demand for sustainable food packaging has led to the development of biobased and biodegradable materials that minimize the environmental impact of conventional plastics. This study introduces an eco-friendly thin film incorporating tannic acid (TA) into a chitosan–poly­(vinyl alcohol) (CPT) matrix to preserve fresh garlic. The film, produced through solvent casting, was thoroughly characterized using several analytical techniques. Fourier-transform infrared spectroscopy (FTIR) and X-ray diffraction (XRD) confirmed strong molecular interactions and increased crystallinity, which enhanced the material’s compatibility and structural integrity. Atomic force microscopy (AFM) revealed a surface roughness of 0.167 nm, and thermogravimetric analysis (TGA) demonstrated thermal stability up to 463°C. The CPT film exhibited notable antimicrobial activity against Staphylococcus aureus, Pseudomonas aeruginosa, and Aspergillus niger. Packaging trials showed that the film effectively extended garlic freshness for 24 days. These findings suggest that the CPT film offers a promising solution for sustainable packaging by combining active food preservation with real-time quality monitoring.

## Graphical abstract

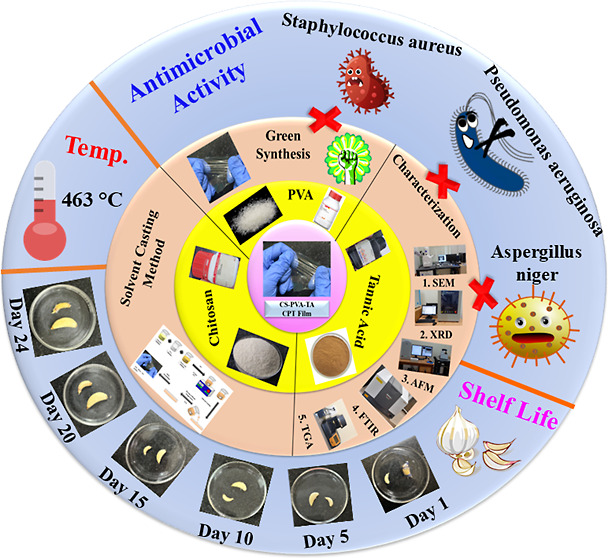

## 1 Introduction

The rapid expansion of the global plastic industry has brought to light a significant environmental issue, white pollution resulting from nonbiodegradable plastic packaging waste. Although food packaging is primarily constructed from petrochemical polymers owing to their cost-effectiveness and protective attributes, their lack of biodegradability has resulted in significant ecological deterioration. This concern has become increasingly urgent in recent years, prompting a deeper investigation into the environmental consequences of using petroleum-based plastics in packaging.^1^ Fresh agricultural products, particularly fruits and vegetables, encounter various challenges related to spoilage throughout the logistics process from harvesting to consumption. These problems are due to physical damage, chemical changes, and microbial contamination.^2^ The main objective of food packaging technology is to create a robust barrier against environmental factors such as microorganisms, moisture, air, and light. This is important to maintain the quality and safety of the food while extending its shelf life.^3^ Developing active packaging material and effective technologies to extend food shelf life is a critical challenge in addressing the issue of food waste reduction. This advancement must also ensure compliance with food safety regulations and meet consumer health needs.^4^

Natural polymers have attracted substantial attention as viable alternatives to petroleum-based plastics. These polymers can biodegrade in the environment through enzymatic or nonenzymatic mechanisms, such as hydrolysis, significantly reducing the environmental problems caused by synthetic polymers.^5^ Natural, biodegradable polymers such as chitosan, cellulose, starch, whey protein, and gelatin have attracted considerable interest in food packaging research.^6^ Among these eco-friendly materials, chitosan is a bright option that has attracted substantial research attention in recent years.^7^ Chitosan and its derivatives are environmentally friendly polysaccharides with numerous beneficial properties. They are nontoxic, compatible with the biological system, and degrade naturally over time. In addition, these materials have antibacterial and antifungal properties and can effectively bind to metal ions.^8,9^ Due to its exceptional film-forming properties, chitosan has been extensively used for potential applications in the food, biomedical, and chemical industries.^10^

Chitosan is produced by the deacetylation of chitin, a major natural polysaccharide known since 1884, and synthesized by various living organisms. Chitin can be obtained from numerous renewable materials mainly as waste from the fishing industry.^11,12^ Therefore, chitosan is an inexpensive and commercially accessible polysaccharide. In its solid form, chitosan is semicrystalline and usually dissolves in dilute organic acids such as acetic, citric, formic, lactic, malic, and tartaric acids. Given these properties, there is growing interest in using it as a material for food packaging film and coating.^11^ Adding PVA to chitosan film significantly improves their mechanical and physical properties, making them suitable for various applications, such as food packaging and wound dressings.^13^ Studies show that combining PVA with chitosan increases tensile strength and elongation at the break due to the formation of hydrogen bonds between the hydroxyl groups of PVA and the amine group of chitosan.^14^ Recently, chitosan-based films have gained significant attention for enhancing food shelf life. In 2020, Liu et al. synthesized a range of chitosan films with varying molecular weights, chitosan content, and glycerol levels, demonstrating their superior performance over conventional polyethylene (PE) films commonly used in the market.^15^ In 2024, He et al. developed chitosan-based coatings (QCTO) incorporating quaternary ammonium salts and tannic acid, which successfully extended the shelf life of bananas, strawberries, and mushrooms by reducing weight loss and inhibiting microbial growth.^16^ Also in 2024, Dăescu et al. introduced biobased films with an anthocyanin analog dye for smart packaging, which provided effective spoilage indicators for pork and chicken through significant color changes, particularly with chitosan–dye and chitosan–PVA–dye films, maintaining performance for up to 30 days.^17^ These innovations underscore the potential of chitosan-based films in food preservation and freshness monitoring.

PVA is a biodegradable, nontoxic synthetic polymer with excellent biocompatibility and is noncarcinogenic. Its superior film-forming capabilities, along with its high hydrophilicity and chemical stability, make it ideal for blending with both synthetic and natural polymers.^18^ PVA is an active polymer distinguished by its unique molecular structure, which contains a high concentration of hydroxyl (−OH) groups. These hydroxyl side chains contribute to PVA’s hydrophilicity and enable it to be modified through the grafting of various functional groups.^19^ The hydroxyl groups also allow PVA to establish hydrogen bonds with other substances, facilitating cross-linking, further improving its mechanical strength and reducing water absorption. Additionally, PVA is well regarded for its exceptional film-forming properties, making it ideal for applications in medicine and food packaging.^20^ PVA exhibits excellent adhesion to various substrates, driven by intermolecular forces, substrate surface properties, and the film formation process. PVA primarily adheres to surfaces through hydrogen bonding and van der Waals forces. Its abundant hydroxyl groups enable strong hydrogen bonding with polar surfaces, such as metals, with bond energies ranging from 10 to 40 kJ/mol, emphasizing the importance of these interactions. Substrate characteristics, including surface roughness and chemical composition, further enhance adhesion. Substrates with a higher hydroxyl content provide more bonding sites for PVA, while surface textures contribute to mechanical interlocking, strengthening the overall bond.^21^ Additionally, drying and solidifying PVA films form a dense polymer network that entangles with the substrate, improving both chemical and mechanical interactions at the interface.^22^ However, while PVA shows strong adhesion, environmental factors like moisture can affect its performance, compromising bond strength under certain conditions. PVA is widely utilized in applications such as controlled drug delivery systems, polymer recycling, film production, and packaging, particularly for creating water-soluble and environmentally friendly materials.

The combination of CS and PVA, both of which have favorable biological activities, can improve the biological properties of the resulting mixed films.^13^ Novel food techniques should combat the main causes of food spoilage and at the same time increase food safety. Therefore, the packaging industry is looking for solutions that offer functional properties such as specific gas barriers, gas or moisture absorbers, UV protection, antioxidant activity, antimicrobial properties solution, and monitoring capabilities to assess product quality. This advanced packaging solution aims to extend the shelf life of food products and thus enables a longer storage period.^23^ Although CS has promising properties for food packaging, it also has certain limitations. In particular, CS lacks sufficient anti-UV, antioxidant, and antibacterial properties. To address these shortcomings, recent studies have investigated incorporating polyphenol compounds into CS. Polyphenols exhibit excellent UV-protective, antioxidant, and antibacterial properties that can improve the performance of chitosan-based packaging materials.^24,25^ In 2024, advancements in food packaging films further highlighted the potential of active materials for extending shelf life. Dong et al. developed PLA three-layer films with PVA-based coatings incorporating chlorogenic acid (CGA) and CGA-functionalized clay, significantly reducing browning and oxidation in fresh-cut apples. These films, particularly with 3 wt % LDHs@CGA, reduced the browning index by 43.8% compared to untreated apples after 36 h.^26^ Similarly, Huang et al. created a smart active packaging using a corn starch/PVA blend with nanosized imidazolate (SIM-1), which demonstrated ammonia sensitivity and antibacterial properties, providing a visual indicator of shrimp spoilage through color changes within 24 h. These innovations underscore the effectiveness of active films in preserving food quality and detecting spoilage.^27^

Tannic acid (TA) is a naturally occurring polyphenolic compound derived mainly from plant sources such as oak galls, chestnuts, and various fruits. It is characterized by its water solubility and weakly acidic properties and consists of a glucose core esterified with several gallic acid units. This unique structure allows TA to undergo significant molecular interactions, including hydrogen bonding, electrostatic interactions, and metal coordination, which are essential for its functionality in various applications.^28^ However, when TA is incorporated into a polymer matrix solely via hydrogen bonding, the resulting composite material does not exhibit significant improvements in mechanical properties. Furthermore, excessive addition of TA can lead to a deterioration in the mechanical properties of the polymer.^29–31^ Tannic acid (TA) is crucial in enhancing the structural integrity and stability of chitosan–PVA films. To avoid TA precipitation within the matrix, its concentration was optimized to remain within solubility limits, ensuring uniform distribution and preventing phase separation. Effective mixing techniques were employed to achieve a homogeneous dispersion of TA, promoting strong hydrogen bonding with the polymer components. Additionally, TA acts as a natural cross-linker, strengthening the polymer network through hydrogen bonding and noncovalent interactions, resulting in improved mechanical properties and a stable three-dimensional structure.^32^ By employing these methods, we effectively produced chitosan–PVA–TA films that exhibit a smooth texture and are free from precipitation. Recent studies have demonstrated the potential of tannic acid (TA) in food packaging to extend shelf life. In 2014, Senna et al. used PVA/CMC/TA blend films to extend the shelf life of bananas from 9 to 19 days.^33^ In 2021, Ma et al. created a TA-shellac film that preserved mangoes for 10 days, improving firmness and reducing weight loss.^34^ In 2023, Park developed TA-coated P3HB-4HB films that kept bananas fresh for 7 days^35^ and Olonisakin et al. reported the synthesis of a PBAT composite film with TA that preserved onions and potatoes for up to 24 days, showing superior preservation over conventional films.^36^

In this study, we developed an innovative chitosan–poly­(vinyl alcohol) (PVA) film incorporating tannic acid (TA) using a simple solution-casting method. This approach offers a more efficient alternative to conventional covalent cross-linking techniques for integrating TA into the polymer matrix, enhancing the mechanical properties of the composite film. The resulting film, designed for food packaging, combines chitosan, PVA, and tannic acid to present improved food preservation capabilities, particularly extending the shelf life of fresh garlic for up to 24 days. Unlike previous studies that relied on nanocomposites for similar purposes, our method achieves superior results without the complexity of nanomaterials. The CPT film demonstrates excellent antimicrobial activity and enhanced mechanical performance, making it a promising candidate for sustainable food packaging solutions.

## 2 Materials and Methods

Poly­(vinyl alcohol) (PVA) with an average molecular weight of 85,000–124,000 and a purity of ≥99% was obtained from Sigma-Aldrich. Chitosan, with a 90% degree of deacetylation, was also sourced from Sigma-Aldrich. Tannic acid (extra pure) was acquired from Loba Chemie, and glacial acetic acid was used without further purification.

## 3 Experimental Section

### 3.1 Preparation of CS–PVA–TA (CPT) Film

The chitosan–poly­(vinyl alcohol)–tannic acid (CPT) composite film was synthesized using a solution casting method. Initially, a 1 wt % poly­(vinyl alcohol) (PVA) solution was prepared by dissolving 1 g of PVA in 100 mL of deionized water. The solution was heated at 90 °C with magnetic stirring at 600 rpm until a clear, homogeneous solution was obtained. Separately, a 1 wt % chitosan solution was prepared by dissolving 1 g of chitosan in 100 mL 2 wt % acetic acid and stirring continuously at 60 °C for 6 h to achieve a homogeneous mixture.^13,37^ Tannic acid was dissolved by mixing 0.5 g of tannic acid powder in 10 mL of deionized water. The three solutions were combined and stirred thoroughly at 60 °C for 45 min. The resulting mixture was poured into Petri dishes and left to dry for 72 h (Scheme 1). Once the solvent evaporated, the dried film was carefully peeled from the Petri dishes.^38^ In selecting the composition for the chitosan–PVA–tannic Acid (CPT) film, we aimed to maximize the unique properties of each component to achieve a balanced and functional material. Chitosan was chosen for its biocompatibility, biodegradability, and inherent antibacterial properties, making it an ideal natural polymer for enhancing the antimicrobial activity of the film. PVA was included due to its excellent film-forming capabilities, mechanical strength, and flexibility, which contribute to the stability and barrier properties of the composite.^39^ The specific concentrations used, 1 g of PVA in 100 mL of water combined with 1 g of chitosan in a 2 wt % acetic acid solution, were selected to ensure optimal viscosity for film formation while maintaining desirable mechanical properties.^40^ Tannic acid 0.1 g in 10 mL was incorporated for its ability to interact with both chitosan and PVA through hydrogen bonding, enhancing mechanical strength and providing additional antimicrobial effects.^41^ The chosen ratio of 3:3:1 of PVA, CS, and TA was based on achieving a synergistic effect that maximizes the functionality of the film without compromising its integrity. This composition is supported by literature indicating that similar ratios yield films with improved mechanical properties and barrier functions.

**Scheme 1:**
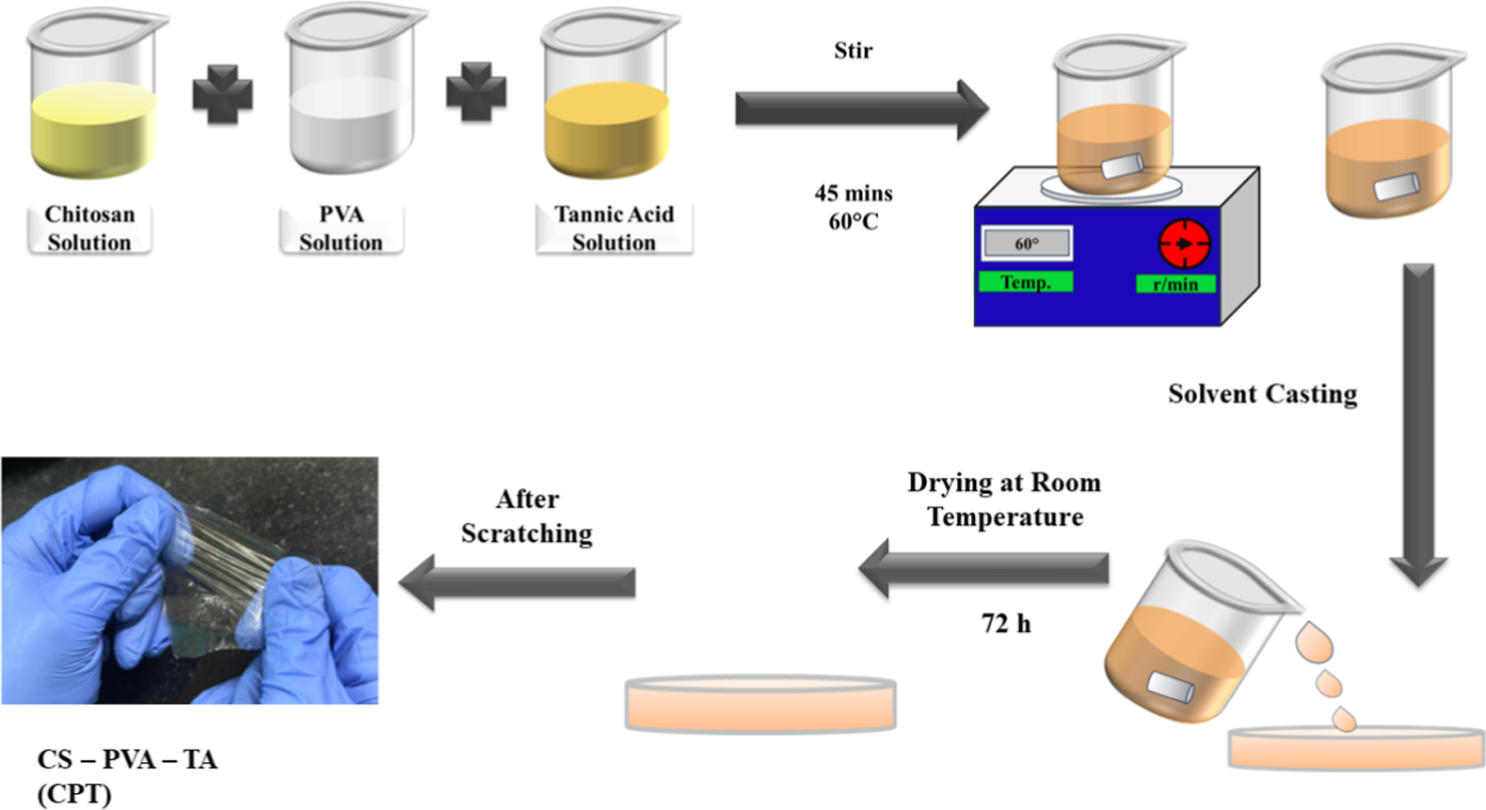
Schematic Representation of Prepared Polymeric CPT Film

### 3.2 Characterization

#### 3.2.1 Fourier-Transform Infrared Spectroscopy

The structure of the CPT film was analyzed using a Shimadzu 8400 FTIR spectrometer, covering a spectral range from 400 to 4000 cm^–1^. This method enabled the detection of functional groups and provided insight into the molecular interactions present in the composite film.

#### 3.2.2 X-ray Diffraction Spectroscopy

The crystalline properties of the CPT film were examined using a Rigaku Miniflex 600 X-ray diffractometer, which operated at a voltage of up to 40 kV and a current of 15 mA. The instrument, fitted with a nickel filter, was set for wide-angle measurements over a 2θ range of 50–130°. The diffraction pattern provided detailed insights into the structural composition of the chitosan–PVA–tannic acid film by analyzing the characteristic peaks in the obtained spectrum.

#### 3.2.3 Electron Spin Resonance

Electron spin resonance (ESR) spectra of the chitosan–PVA–tannic acid (CPT) film were recorded using a JEOL JES-FA200 ESR Spectrometer (Japan), operating in the X-band (8.75–9.65 GHz). The instrument offers high sensitivity (7 × 10^9^ spins/0.1 mT) and a resolution of 2.35 μT, ensuring precise detection of spin interactions. Measurements were conducted on the top surface of the polymeric film to analyze its structural and electronic properties. The ESR characterization provided valuable insights into the crystallinity and molecular interactions within the CPT matrix, which were further correlated with other analytical techniques.

#### 3.2.4 UV–Visible Spectroscopy

The UV–visible analysis of the samples was carried out using a UV–visible spectrophotometer. UV–visible double beam spectrometer 2201 was used to obtain the spectra of the samples. Information was recorded in the absorbance mode in the wavelength choice of 200–800 nm.

#### 3.2.5 Surface Morphology

The surface characteristics of the CPT film were initially examined using a scanning electron microscope (SEM), specifically the EVO MA18 model equipped with an Oxford EDS (X-act) for elemental analysis. The SEM analysis was performed at a magnification of 1000× with a depth of focus suitable for observing fine surface details, an aperture size of 100 μm, and a working distance of 10 mm, corresponding to a depth of field around 40 μm. The study was conducted under controlled conditions in an air-conditioned environment (21–24 °C) with relative humidity below 60%, and nitrogen gas of 99.99% purity was used at a pressure regulated between 0 and 3 bar. Further, field emission scanning electron microscopy (FESEM) was performed using the FESEM [7610FPLUS, Jeol, Japan] to obtain a more detailed surface morphology analysis. This technique provided higher-resolution imaging, enabling enhanced visualization of the finer surface features of the composite film.

#### 3.2.6 Thickness

The thickness of the film was determined using a calibrated Plunger dial digital gauge (Mitutoyo no. 547–401, Tokyo, Japan), ensuring precise and reliable measurements. The procedure was conducted under controlled conditions to maintain consistency and minimize errors, providing reproducible data on film thickness for scientific analysis.

#### 3.2.7 Tensile Properties

A universal testing machine, Shimadzu EZ-SX, was utilized to evaluate the mechanical properties of the films, including their stress–strain behavior under tensile loading. The machine, manufactured by Shimadzu, has a load capacity of 500 N (approximately 110 lbs) and operates at testing speeds ranging from 0.01 to 500 mm/min, with a stroke length of 800 mm (approximately 31.5 in.). Tensile tests were conducted at room temperature, and six samples were tested for each film formulation. From the resulting stress–strain curves, key mechanical parameters such as Young’s modulus (*E*), tensile strength (TS), and elongation at break (EAB) were determined.

#### 3.2.8 Thermal Gravimetric Analysis

The thermal behavior and decomposition profile of the CPT film were assessed using thermogravimetric analysis (TGA) with a Diamond TG/DTA Instruments. The study was conducted from 30 to 800 °C at a heating rate of 10 °C/min in a nitrogen atmosphere to prevent oxidation. This approach provided key insights into the thermal stability and degradation characteristics of the CPT film.

#### 3.2.9 Atomic Force Microscopy

Atomic force microscopy (AFM) was used to analyze the surface roughness of the CPT film using a Bruker Innova IB342 and provided detailed insights into its structural characteristics and material behavior.

#### 3.2.10 Antimicrobial Activity

To evaluate the sustainability of the CPT film as an antimicrobial agent for food packaging applications, an antimicrobial simulation test was performed utilizing the agar cup methodology, an established technique for evaluating antimicrobial efficacy. This methodology requires the preparation of an agar medium inoculated with microorganisms that exhibit sensitivity to the antimicrobial agents under investigation, subsequently distributed into Petri dishes. Solutions of the CPT film, along with reference antimicrobial compounds, are administered onto the agar surface, either in specified cavities or on paper discs, followed by a controlled incubation period to facilitate microbial proliferation. Following the incubation period, the diameters of the inhibition zones are carefully measured to evaluate the efficacy of the antimicrobial agents, with statistical analyses employed to determine their strength and significance. The application of multiple replicates for each test enhances the reliability and reproducibility of the results. This standardized methodology offers an efficient and durable framework for the evaluation of the antimicrobial characteristics of the CPT film, thereby signifying its potential utility in food packaging to improve food safety and prolong shelf life.

#### 3.2.11 Shelf Life

The shelf life examination was conducted to measure the efficacy of the CPT film on fresh garlic, selected for its translucent appearance and procured from a local supermarket in Manipal, Karnataka, India. A garlic clove was wrapped in the carefully prepared CPT film and securely fastened with thread to ensure adequate sealing. Systematic visual evaluations were performed to detect changes in the garlic stored at ambient temperature over a specified duration. Observations focused on variables such as color, texture, and overall freshness, allowing for a comprehensive analysis of the CPT film’s ability to extend the shelf life of fresh garlic. This methodology provides valuable interpretations into the potential use of CPT film in food packaging, particularly in enhancing the durability and quality of perishable goods.

## 4 Results and Discussion

### 4.1 Fourier-Transform Infrared Spectroscopy

The characteristic peaks representing the bending and stretching vibrations of functional groups in the pure chitosan, PVA, tannic acid, and the synthesized CPT films illustrate significant chemical interactions within these materials as shown in Figure 1. The FTIR analysis of the pure chitosan film corresponds to various functional groups that indicate the chemical structure and interactions within the material. The peaks at 3423.80 and 3268.67 cm^–1^ are associated with (O–H) and (N–H) stretching vibrations, respectively, indicating the presence of hydroxyl and amine groups. The peak at 2865.66 cm^–1^ is indicative of (C–H) stretching, while the sharp peak at 1639.39 cm^–1^ corresponds to carbonyl (C=O) stretching of amides. Other notable peaks include 1540.08 cm^–1^ for (N–H bending), 1375.92 cm^–1^ for (C–H bending), and 1067.30 cm^–1^ (C–O stretching),^42,43^ which collectively suggest strong hydrogen bonding and structural integrity within the chitosan matrix. Moreover, in pure PVA film, a peak at 3255.54 cm^–1^, indicates (O–H) stretching, characteristics of hydroxyl groups,^44^ while peaks at 2941.17 cm^–1^ and 2849.24 cm^–1^ are attributed to (C–H) stretching vibration from methylene (−CH_2_) and methyl (CH_3_) groups, respectively.^45^ The peak at 1641.86 cm^–1^ corresponds to (C=O) stretching, suggesting the carbonyl group’s presence. The peaks at 1425.99 and 1325.85 cm^–1^ relate to (C–H) bending vibration, reinforcing the presence of alkyl chains in the polymer matrix.^46^ Additionally, the peak at 1144.45 cm^–1^ is linked to (C–O) stretching, indicative of ether or alcohol functionalities,^44^ while the peaks at 1084.54 and 917.92 cm^–1^ further support (C–O) linkage and may indicate specific saccharide structures or other configurations.^47^ Finally, the peaks at 844.04 cm^–1^ may relate to the out-of-plane bending vibration of (C–H) bonds in cyclic structures.^46^ In Tannic acid, the absorption band at 3383.58 & 3244.87 cm^–1^ indicates (O–H) stretching, characteristics of strong hydrogen bonding interactions. The peak at 1708.34 cm^–1^ is attributed to (C=O) stretching, indicating the presence of carbonyl groups, likely from ester or carboxylic acid functionalities.^48^ The peak at 1606.56 cm^–1^ is associated with (C=C) stretching in aromatic rings, while the peak at 1531.87 cm^–1^ corresponds to (C=C) stretching, reinforcing the aromatic character of tannic acid. The peak at 1447.33 cm^–1^ is related to (C–H) bending vibrations, typical for methyl and methylene groups in the structure. Peaks at 1312.72 and 1190.42 cm^–1^ are attributed to (C–O) stretching, indicating ether or alcohol functionalities, and reinforcing the polyphenolic nature of tannic acid. Whereas chitosan–PVA film 3383.58 cm^–1^ indicates (O–H) stretching, characteristics of hydroxyl groups suggest hydrogen bonding interaction. Similarly, the peak at 3244.87 cm^–1^ also corresponds to (N–H) stretching, confirming the amine functionality in chitosan.^49^ The absorption band 2905.88 cm^–1^ corresponds to (C–H) stretching vibrations, indicative of aliphatic hydrocarbons’ presence of polymer chains.^50^ 1771.54 cm^–1^ is associated with (C=O) stretching, likely from ester or carbonyl functionalities present in film.^51^ The peak at 1643.50 cm^–1^ corresponds to the amide I band, indicating (C=O) stretching from amide in chitosan,^52^ while the peak at 1549.11 cm^–1^ is attributed to the amide II band, reflecting (N–H) bending and (C–N) stretching.^53^ The peak at 1415.32 cm^–1^ relates to (C–H) bending vibrations, typical for methyl and methylene groups. Peaks at 1316.82 and 1147.74 cm^–1^ are attributed to (C–O) stretching, indicating ether or alcohol functionalities that are common in both PVA and chitosan.^52^ The peak at 1087 cm^–1^ also corresponds to (C–O) stretching, further supporting the presence of hydroxyl and ether linkages. The peaks at 1015.59, 894.11, and 845.69 cm^–1^ are associated with additional (C–O) stretching and out-of-plane bending vibrations of C–H bonds in aromatic systems,^52^ while the peaks at 768.53 and 653.62 cm^–1^ may indicate skeletal vibrations or other structural features within the polymer matrix, including potential interactions between chitosan and PVA. Finally, the peak at 558.41 cm^–1^ could be related to skeletal vibrations or cross-linking interactions within the composite film.^53^ In the synthesized CPT film, the spectrum exhibits several significant peaks, and the broadness, and absorbance at 3283.40 cm^–1^ corresponds to (O–H) stretching vibrations of hydroxyl groups, indicating the presence of alcohols and phenolic compounds. The asymmetric and symmetric C–H stretching vibration of aliphatic hydrocarbons, typical in both chitosan and PVA^54,55^ are present at 2921.11 and 2851.68 cm^–1^. The absorption peak at 1708.96 cm^–1^ indicates (C=O) stretching, which arises from the carbonyl groups in tannic acid or the ester linkage formed while blending.^56^ The 1648.31 cm^–1^ peak associated with amide I band (C=O) stretching modes from chitosan confirms its presence in the film. The band at 1550.95 cm^–1^ represents (N–H) bending vibrations related to the amide groups in chitosan.^54,57^ The absorption peaks at 1448.81, 1379.38, and 1309.16 cm^–1^ are associated with (C–H) bending vibration, (CH_3_) groups, and (C–N) stretching vibrations respectively, which are characteristics of organic compounds including chitosan and PVA.^58,59^ (C–O) stretching vibrations, indicating the presence of hydroxyl groups from PVA and tannic acid are represented by 1216.59 and 1142.38 cm^–1^ absorption peaks.^56^ The bands at 844.73 and 776.10 cm^–1^ correspond to out-of-plane bending vibrations of (C–H) bonds and specific bending modes in the polymer structure, respectively.^60^ This analysis reveals significant interactions between chitosan, PVA, and tannic acid, highlighting the strong interactions and the formation of a complex network within the composite films, enhancing their potential for application in food packaging.

**Figure 1:**
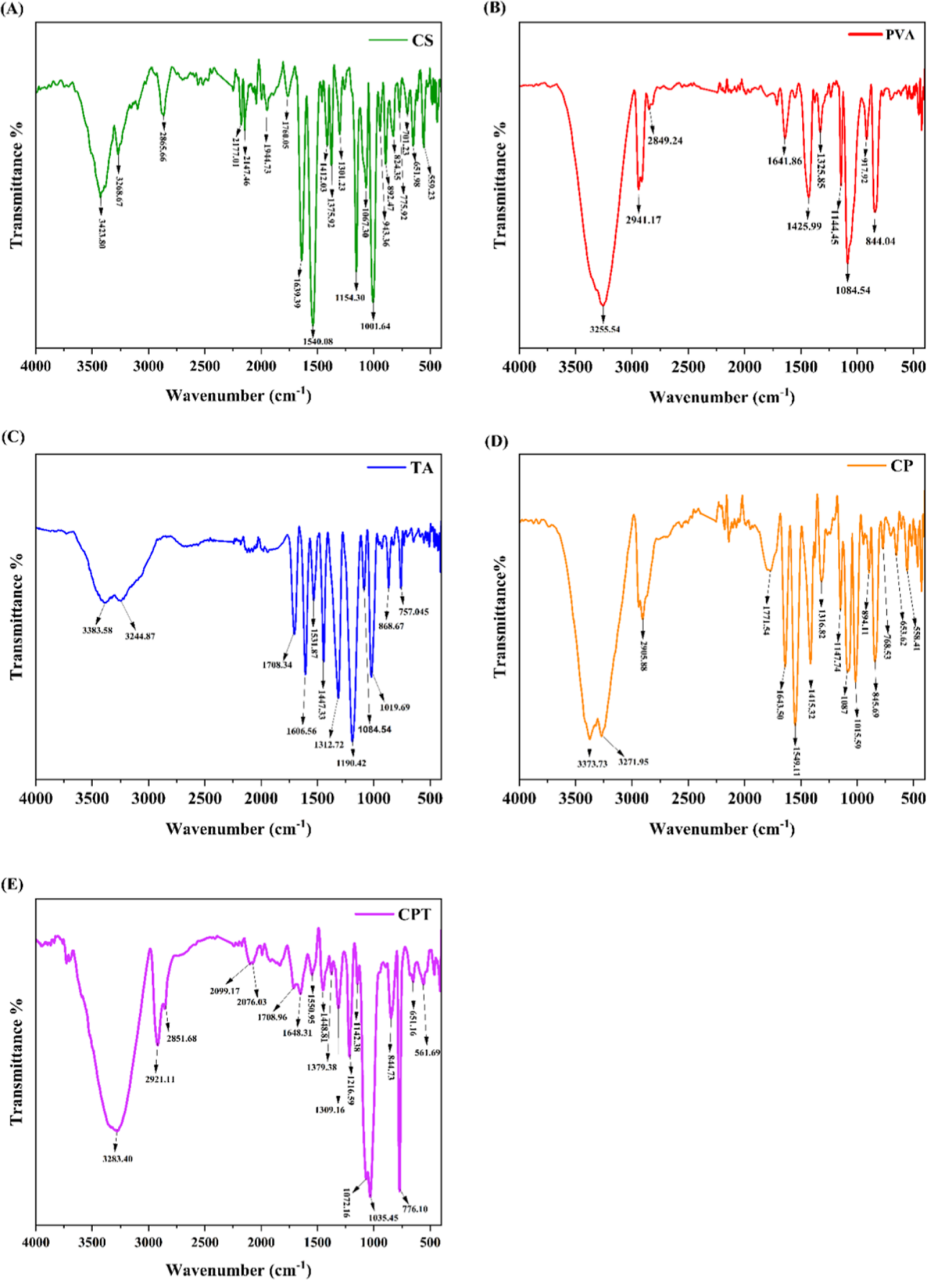
Fourier-transform infrared spectroscopy analysis of (A) chitosan film, (B) PVA film, (C) tannic acid, (D) chitosan–PVA film, (E) CPT film.

### 4.2 X-ray Diffraction Spectroscopy

The X-ray diffraction (XRD) results for the chitosan film, PVA film, pure tannic acid, and CPT film provide important insights into their structural properties, as represented in Figure 2. Chitosan exhibits a degree of partial crystallinity, as indicated by reflections at 10 and 20° at a plane (110), corresponding to crystalline structures I and II.^61^ These reflections are attributed to the ordered chain architecture of the chitosan, which highlights the role of hydrogen bonding between the polymer chains in enhancing its overall crystallinity. The PVA displays semicrystalline characteristics, as evidenced by subdued diffraction peaks at 11.4 and 22.9°, associated with crystal planes (101), (100), and (200) in the reported literature.^62,63^

**Figure 2:**
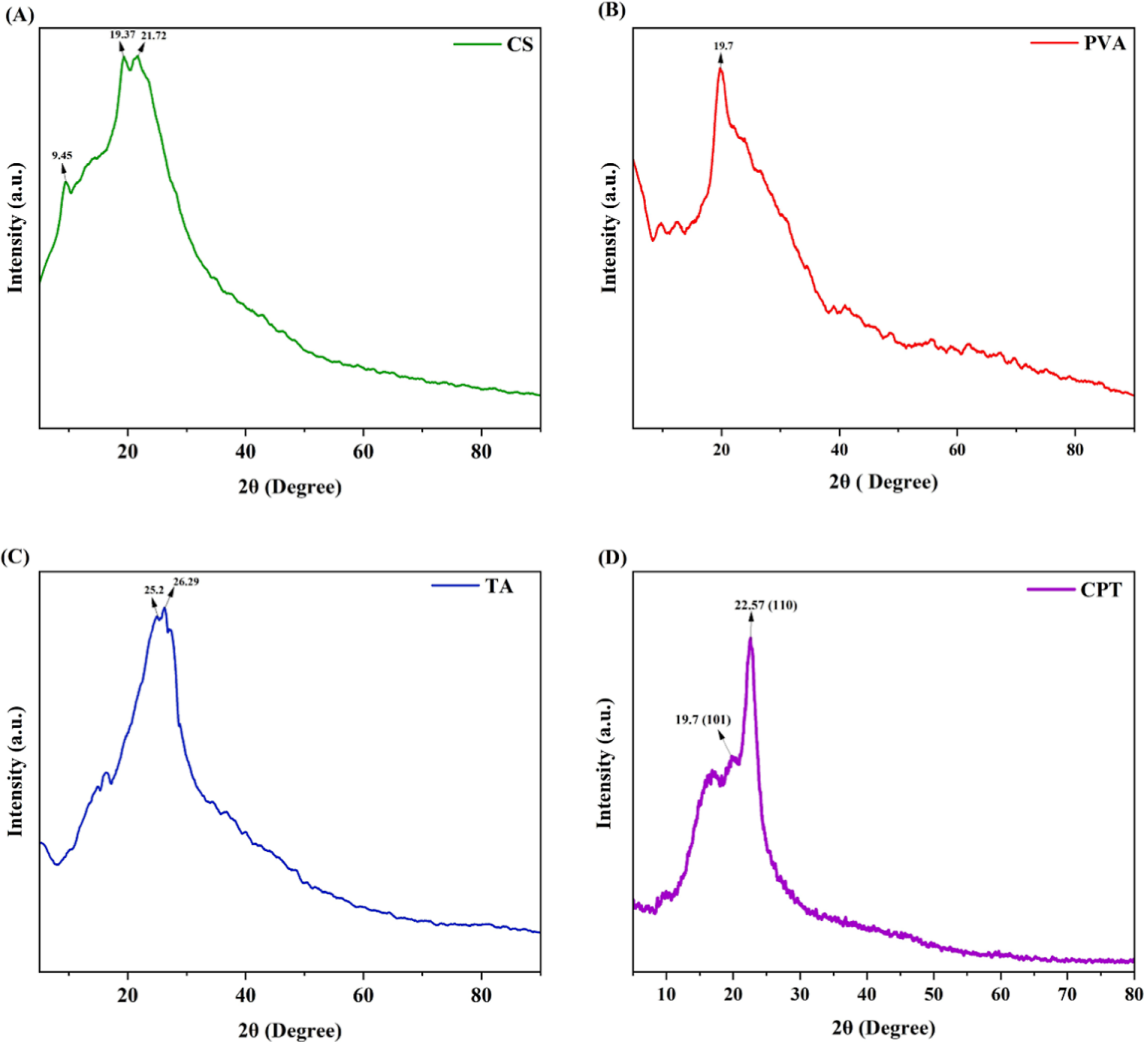
XRD diffractograms showing the crystalline structures of (A) chitosan (CS) film, (B) poly­(vinyl alcohol) (PVA) film, (C) pure tannic acid (TA), and (D) chitosan–PVA–tannic acid (CPT) film.

In the synthesized chitosan film, peaks at 9.45, 19.37 and 21.72° reflect its crystalline region contributing to mechanical strength, while the peak at 19.7° (101) in PVA film confirms its semicrystalline nature, which improves film strength. The CPT film shows a peak at 22.57° (110) for chitosan and a peak at 19.7° (101) for PVA, indicating a change in crystalline configuration due to interactions between the polymeric components.^60^ The integration of tannic acid (TA) with PVA does not significantly change the peak positions, indicating that the interactions between the hydroxyl groups of chitosan and PVA with the tannic acid are crucial for the improvement of crystallinity. The formation of intermolecular hydrogen bonds between the tannic acid and the chitosan–PVA polymer matrix is crucial for increasing the peak intensity. This phenomenon emphasizes the critical role of hydrogen bonding in maintaining the structural integrity and crystallinity of the composite films, which affects their potential applications in various fields. Pure tannic acid (commercially available), with peaks at 25.2, and 26.29°, exhibits a crystalline structure that disappears upon blending, further emphasizing its integration into the amorphous matrix of the composites.^64^ The lack of peaks for tannic acid in the CPT film suggests it may be uniformly dispersed, contributing to the film’s properties without forming distinct crystalline structures.^60^ In contrast, while the blending of these materials enhances certain properties, it may also lead to a loss of individual crystalline characteristics, which can be a drawback in applications requiring high structural integrity.

The average crystallite size (*D*) was determined using the Scherrer equation. The measured values of [2θ, θ, and the full width at half-maximum (fwhm)]. For a 2θ value of 22.67°, the corresponding θ value is 11.335°, with a fwhm of 1.72. This analysis yielded a crystallite size (*D*) of about 2.53 × 10^–10^ meters when θ is expressed in degrees, and about 8.58 × 10^–11^ meters when θ is expressed in radians. The calculated crystallite size (*D*) provides important insights into the structural properties of the CPT film.

The Debye–Scherrer equation is used to calculate the crystalline size as follows

$$D=\frac{K\lambda }{\beta \;\cos\;\theta }$$

where *D* represents the crystalline size, *K* represents the Scherrer constant (0.98), λ denotes the wavelength (1.54), and β denotes the full width at half-maximum (fwhm).

### 4.3 Electron Spin Resonance

The electron spin resonance (ESR) spectrum of the chitosan–PVA–tannic acid (CPT) film exhibits a Lorentzian shape characteristic of polymeric materials, as shown in Figure 3. The extracted parameters, including the *g*-value, line width (Δ*H*), and signal intensity, provide insights into the film’s structural properties.

**Figure 3:**
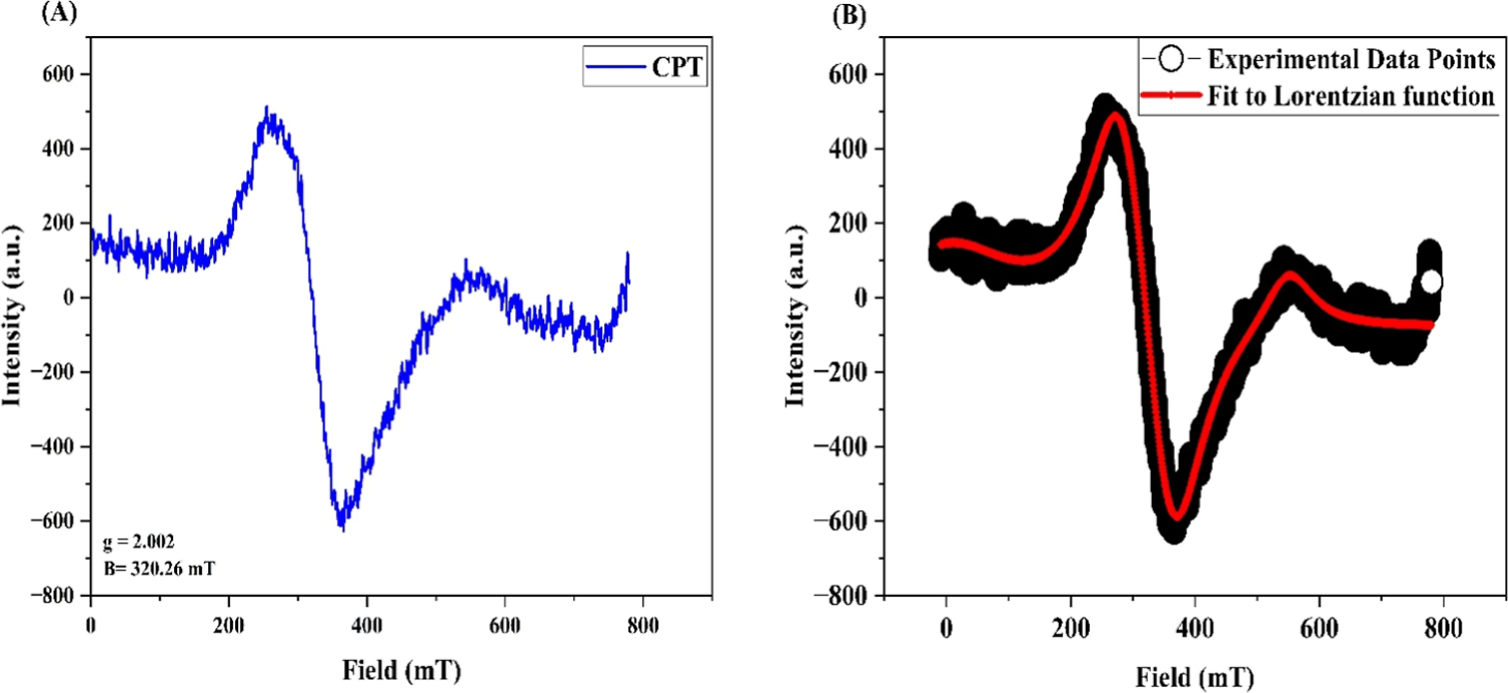
ESR spectra of (A) CPT film and (B) Lorentzian fit of the CPT film.

The g-value is calculated using the following equation

$$g=\frac{h\nu }{\mu_{\beta }B}$$

where *h* is Planck’s constant (6.626 × 10^–34^ J·s), ν is the microwave frequency (9.4495 GHz = 99.4495 × 10^9^ Hz), μ_β_ is the Bohr magneton (9.274 × 10^–24^ J/T), and *B* is the magnetic field at resonance (320.26 mT = 0.32026 T) on the *x*-axis of the graph. Plugging in these values, the calculated *g*-value is approximately 2.002, close to the free electron *g*-value (2.0023). This suggests that the unpaired electrons in the sample are in a relatively free or weakly interacting environment, typical of amorphous regions in polymers.

The line width (Δ*H*) of the ESR signal provides further evidence of the structural characteristics. A narrower line width indicates higher molecular mobility, which is typically associated with lower crystallinity and a more flexible polymer matrix. The ESR data, including the *g*-value (∼2.002) and narrow line width, suggest that the CPT film exhibits significant amorphous character, aligning with the known structural behavior of chitosan and PVA-based films.^65,66^

The ESR and XRD results provide complementary insights into the CPT film’s structure. XRD analysis confirms partial crystallinity due to polymer interactions and hydrogen bonding, while the absence of distinct tannic acid peaks suggests its uniform dispersion within the amorphous phase. While XRD detects localized crystalline regions, ESR confirms a predominantly amorphous structure, as indicated by the observed spectral parameters. This structural balance enhances mechanical strength through small crystalline domains while maintaining flexibility due to the amorphous matrix.

### 4.4 UV–Visible Spectroscopy

The UV absorption spectra of chitosan film, PVA film, and pure tannic acid exhibit distinct characteristics as shown in Figure 4. Each of these materials demonstrates unique optical properties in the UV region, which can be attributed to their molecular structure and interactions. Chitosan shows significant UV absorption, particularly in the range of 200–300 nm, due to its amino and hydroxyl functional groups, which can absorb UV light effectively. PVA generally displays lower UV absorption, primarily in the range of 200–250 nm.^67^ Tannic Acid is known for its strong UV absorption capabilities, particularly in the 250–300 nm range, due to its polyphenolic structure, which allows for effective UV shielding.^68^

**Figure 4:**
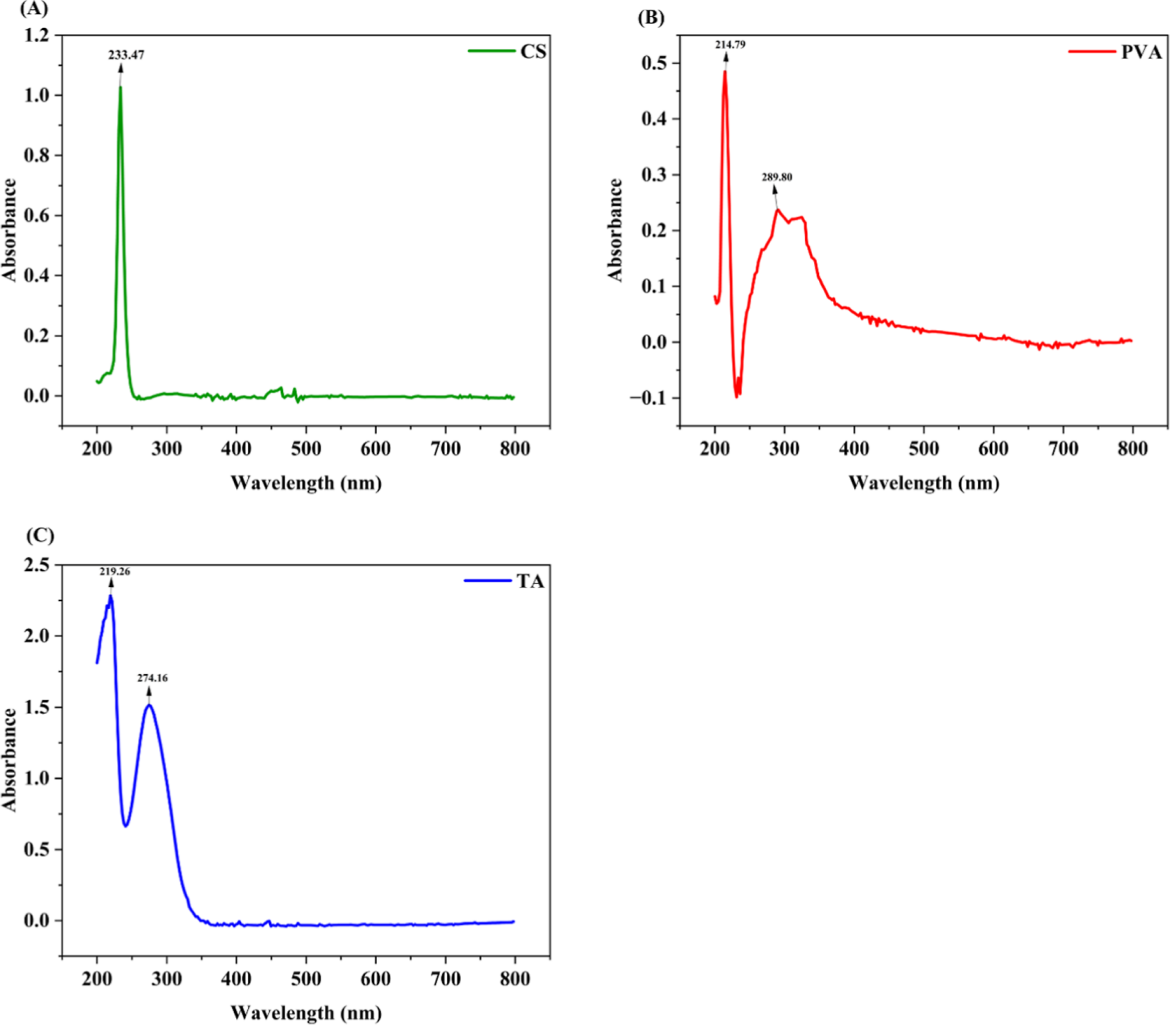
UV absorption spectra of (a) chitosan, (b) poly­(vinyl alcohol) (PVA), and (c) tannic Acid, highlighting their distinct optical absorption characteristics in the UV region.

In our study chitosan exhibits a sharp peak at 233.47 nm, which corresponds to π→π* transition of the C=O and C=N bonds.^69,70^ Whereas PVA demonstrates a sharp at peak 214.79 nm is attributed to the π→π* transition of the C=O groups in PVA, while the broader peak at 289.80 nm may indicate intermolecular interaction or hydrogen bonding.^71^ The peak observed at 219.26 nm indicates the role of tannic acid’s phenolic structure in its UV absorbance. The π → π* transition is associated with the phenolic groups present in tannic acid. The presence of multiple hydroxyl groups in its structure enhances its ability to absorb UV light, which is a characteristic feature of polyphenolic compounds. A study reported that tannic acid exhibits a characteristic peak around 213 nm, which closely aligns with the observed peak at 219.26 nm in this analysis, thereby confirming its association with the phenolic structure of tannic acid.^72^ The second peak, observed at 274.16 nm, corresponds to the extensive aromatic structure of tannic acid, highlighting its efficient UV light absorption capabilities. This peak is attributed to the extended conjugation within the aromatic rings, which enhances light absorption in the UV region. These characteristics underscore tannic acid’s potential for use in UV-blocking applications.^73,74^

### 4.5 Surface Morphology

SEM analysis was employed to analyze the film’s microstructure, focusing on voids, layer homogeneity, and surface smoothness as depicted in Figure 5. The SEM images revealed no discernible clustering, fractures, or pores across all films, suggesting successful dispersion of cross-linkers within PVA microdomains and favorable component compatibility. This phenomenon can be attributed to the abundance of (−OH) groups in PVA, Chitosan, and Tannic acid. SEM analyses show that as a result, the cross-linked PVA film exhibits a continuous matrix with good structural stability.^37,75,76^

**Figure 5:**
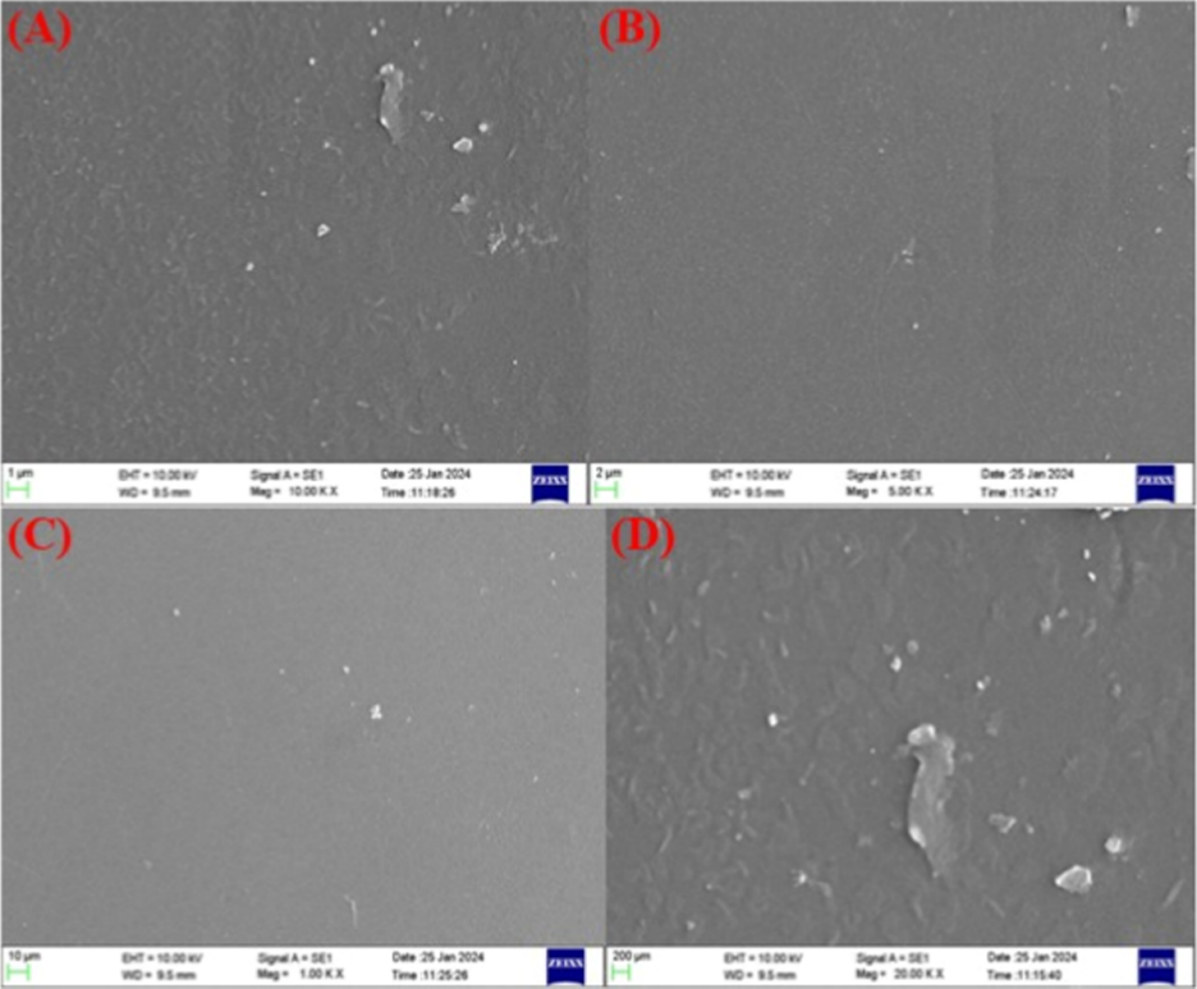
SEM images of the CPT film with scale bars of (A) 1, (B) 2, (C) 10, and (D) 200 μm.

The film’s morphological characterization was carried out using field emission scanning electron microscopy (FESEM). This method offers critical insights into structural features, including the presence of voids, the uniformity and homogeneity of the composite material, the formation of aggregates, the distribution of nanoparticles within the continuous matrix, and the potential alignment or orientation of the nanoparticles.^77^ The observations were performed on the surface of the chitosan–PVA film (without tannic acid) after the synthesis. Figure 6A–D show the film’s Fe-Sem micrograph. The smooth surface morphology of the blended film suggests a uniform dispersion within the blend matrix. This observation can be attributed to the formation of hydrogen bonding interactions between the amino and hydroxyl groups of chitosan and the hydroxyl groups of poly­(vinyl alcohol).^78^

**Figure 6:**
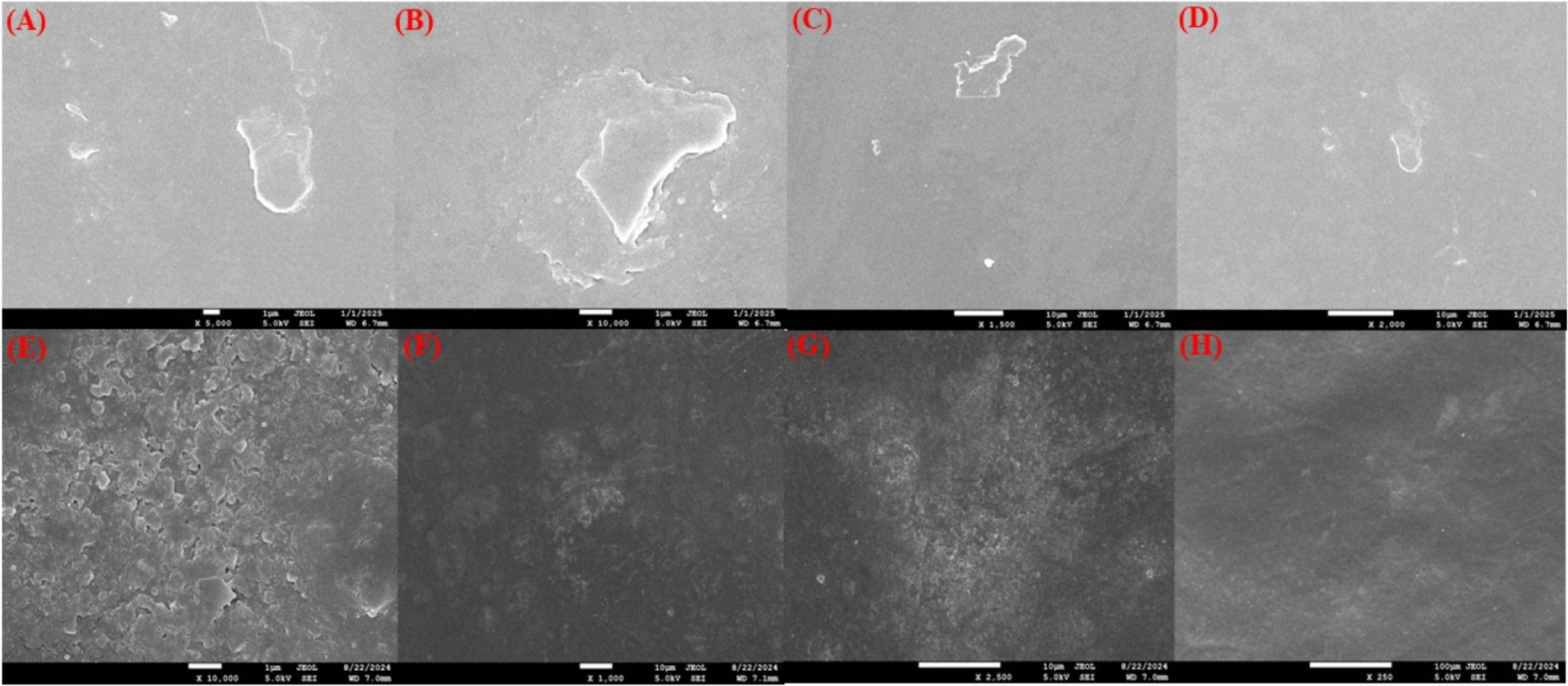
FE-SEM images illustrating the surface morphology and microstructural characteristics of the CP film at magnifications of (A) 5000×, (B) 1000×, (C) 1500×, and (D) 2000×, and the CPT film at magnifications of (E) 10,000×, (F) 1000×, (G) 2500×, and (H) 250×.

Figure 6E–H show the FESEM images of the tannic acid-incorporated chitosan–PVA (CPT) film. At lower magnifications, the film surface appears smooth and uniform, indicating a relatively homogeneous distribution of components at the macroscopic level. However, at higher magnifications, distinct bulge-like structures become apparent. These bulges are likely due to the incorporation of tannic acid into the polymer matrix, which can induce cross-linking through hydrogen bonding and interactions with the chitosan and PVA chains. This cross-linking enhances the mechanical strength and stiffness of the film but may also create localized stress concentrations, leading to the formation of surface irregularities or bulges.^79,80^ Additionally, the tannic acid may cause phase separation or aggregation due to its interaction with the polymer matrix, further contributing to the observed surface morphology. As the concentration of tannic acid increases, these interactions become more pronounced, potentially causing greater stiffness and resulting in stress points that manifest as surface bulges, particularly under conditions such as moisture absorption or thermal expansion. These observations highlight the complex interplay between the components of the CPT film and their impact on its structural properties and morphology.^54,81^

**Figure 7:**
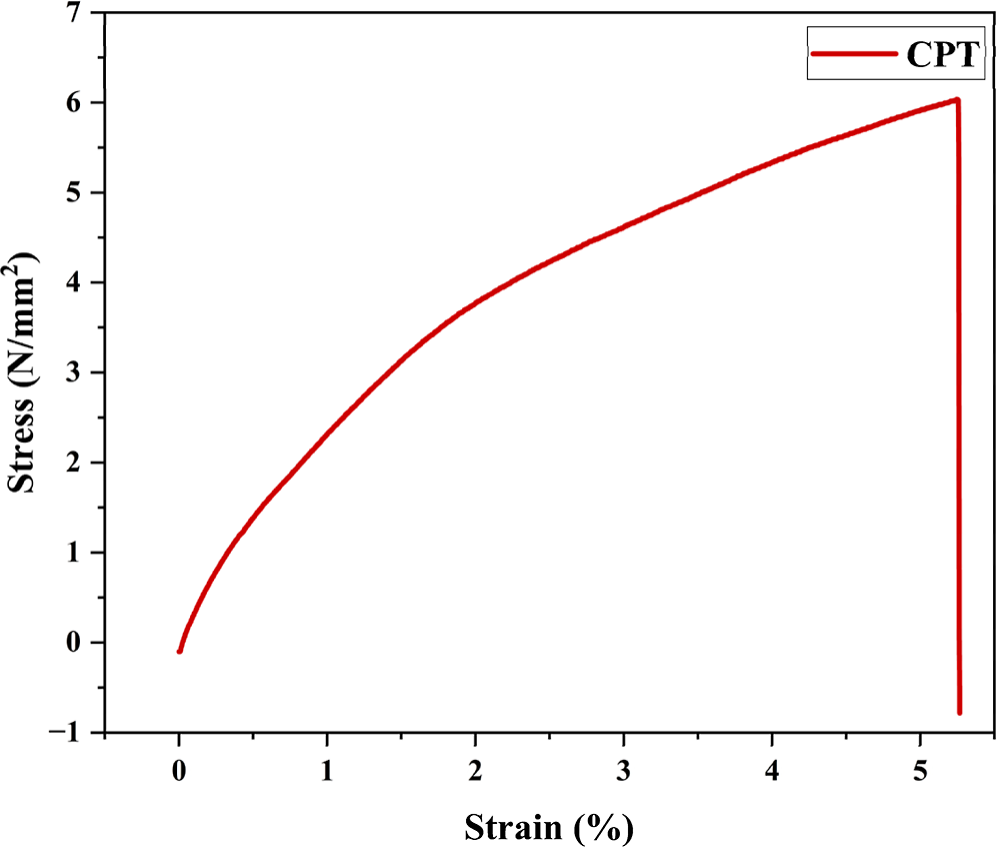
Tensile strength graph of chitosan–PVA–tannic acid (CPT) film.

### 4.6 Thickness

The appearance of the packaging plays an important role in the consumer’s perception of the food it contains. According to the literature, the standard thickness of pure poly­(vinyl alcohol) (PVA) films is around 0.59 mm, while pure chitosan (CS) films have a much greater thickness of around 46.11 mm. For chitosan–poly­(vinyl alcohol) (CS–PVA) laminated films, the thickness ranges from 44.81 to 59.82 mm.^82^ These thickness variations emphasize this parameter’s importance for effective food preservation and protection, which in turn affects consumer acceptance and satisfaction. In this study, the thickness of the synthesized chitosan–poly­(vinyl alcohol)–tannic acid (CPT) film was found to be 0.03 mm, a significantly thinner thickness, suggesting greater flexibility and ease of handling. This can further improve the impressive and functional properties of the packaging. In the context of packaging, film thickness is an important parameter that significantly influences the functional properties of the material, independent of consumer acceptance and satisfaction. From a scientific perspective, the thickness of a film directly influences its mechanical strength and overall performance in packaging applications. Thicker films generally provide better barriers against oxygen and moisture, which are critical for preserving the freshness and quality of perishable products.^83^ For instance, a suitable thickness can extend the shelf life of food items by minimizing spoilage and maintaining optimal conditions during storage and transportation. Additionally, the thickness of the film affects its puncture resistance and durability, which are vital during handling and shipping.^84^ Therefore, optimizing film thickness is crucial not only for ensuring product integrity but also for enhancing overall packaging performance in various applications.^85^ The thickness of the films has been compared with values reported in the existing literature (Table 1).

**Table 1:** Evaluation of the Impact of Varying Polymeric Film Thicknesses on Food Preservation Effectiveness

S. no	author	year	polymeric film	thickness (mm)	references
1	our paper	2024	CPT	0.03	
2	He et al.	2024	QCTO	0.003	^16^
3	Dong et al.	2024	PVA-CGA/PVA	0.0043	^26^
			LDHs@CGA/PVA	0.0042	
4	Huang et al.	2024	CS/PVA/SIM-1	0.06	^27^
5	Park et al.	2023	pristine P3HB-4HB	0.08	^35^
			plasma treated P3HB	0.0638	
			TA-coated P3HB-4HB	0.0659	
6	Olonisakin et al.	2023	PBAT/ESO-Lig-Ta	0.09	^36^
7	Ferri et al.	2023	PHBV/tannin	0.08	^86^

### 4.7 Tensile Strength

The tensile strength of the films is significantly dependent on the structure and the interactions between the components in the polymeric matrix as shown in Figure 7. In this study, incorporating chitosan and tannic acid into the PVA matrix resulted in a tensile strength of 6.030 MPa. This value reflects the combined effect of chitosan’s reinforcing capability and tannic acid’s cross-linking potential. The elongation at break of the chitosan–PVA–tannic acid film was 5.263%, indicating a moderate level of flexibility compared to other films developed in similar studies. Young’s modulus was also measured at 1.909, demonstrating an improvement in stiffness due to the synergistic interaction between chitosan and tannic acid. This interaction likely promotes a more compact and rigid network structure within the film. The reduction in elongation at break could be attributed to the restricted mobility of the polymer chains, caused by the cross-linking effect of tannic acid and the reinforcing action of chitosan.

### 4.8 Thermal Gravimetric Analysis

The thermal behavior of the pure chitosan film, PVA films, tannic acid, and synthesized CPT film was thoroughly evaluated using thermogravimetric analysis (TGA) as depicted in Figure 8. The TGA curve of pure chitosan exhibits two distinct weight loss stages between 29.04 and 350.34 °C. The initial stage, occurring from 29.04 to 148.05 °C, corresponds to the loss of water molecules, resulting in a weight reduction of approximately 15.95%. The primary degradation of chitosan begins at 148.05 °C and concludes around 350.34 °C, leading to an additional weight loss of about 16.62%.^87^ The thermogravimetric analysis (TGA) curve of PVA (Curve A) reveals three distinct stages of weight loss. The first stage, attributed to moisture release, shows a weight loss of approximately 10.61% within the 30.59–148.06 °C temperature range. Beyond 148.06 °C, no significant weight loss is observed. The second stage exhibits rapid degradation, with a substantial weight loss of about 69.07% between 239.19 and 358.25 °C. Finally, the third stage, from 432.14 to 464.86 °C, shows a small weight loss of 4.52%, which can be ascribed to the decomposition and degradation of the PVA film.

**Figure 8:**
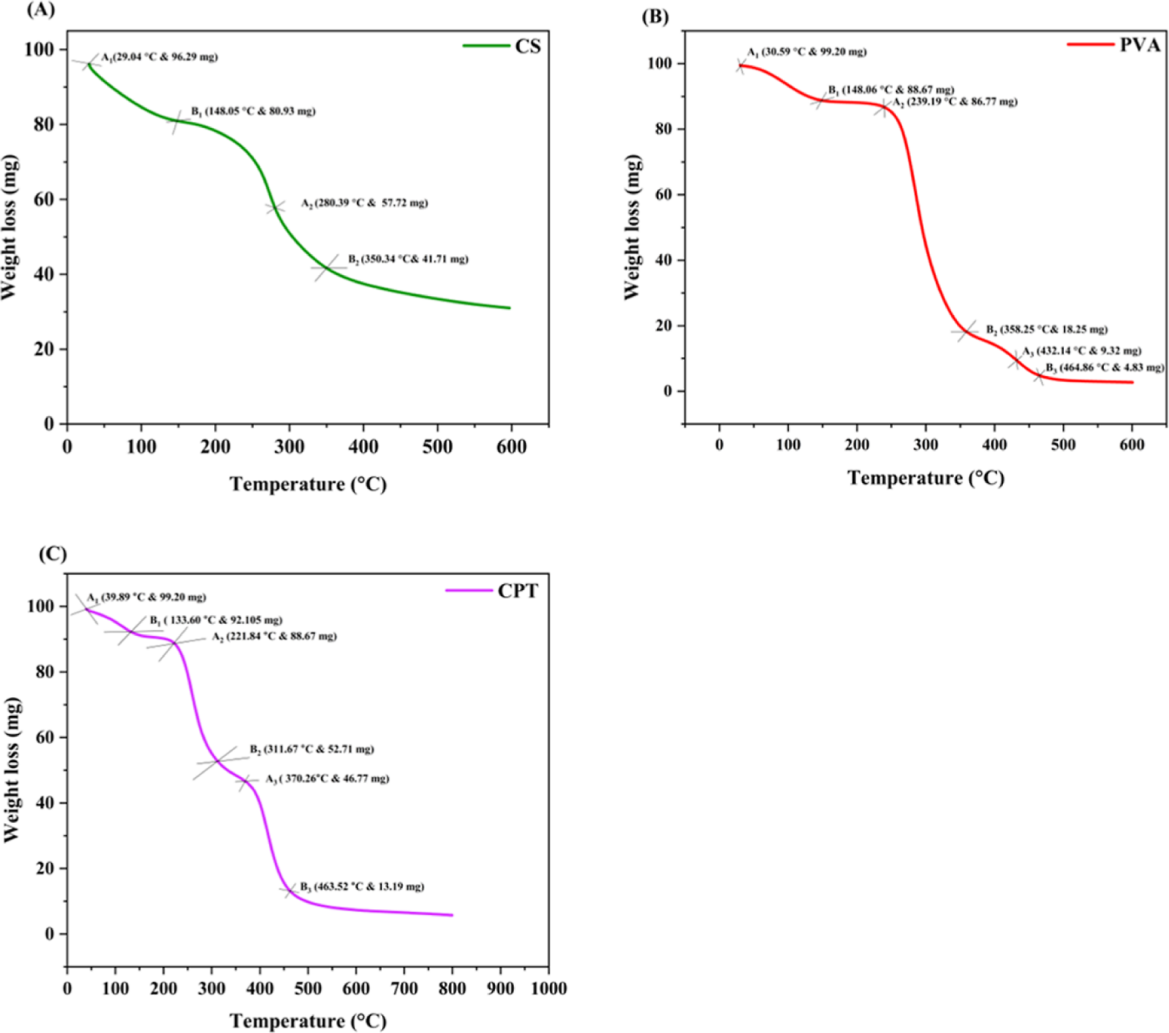
TGA thermogram of (A) chitosan film, (B) PVA film, (C) CPT film depicting thermal degradation characteristics and weight loss across different temperature ranges.

**Figure 9:**
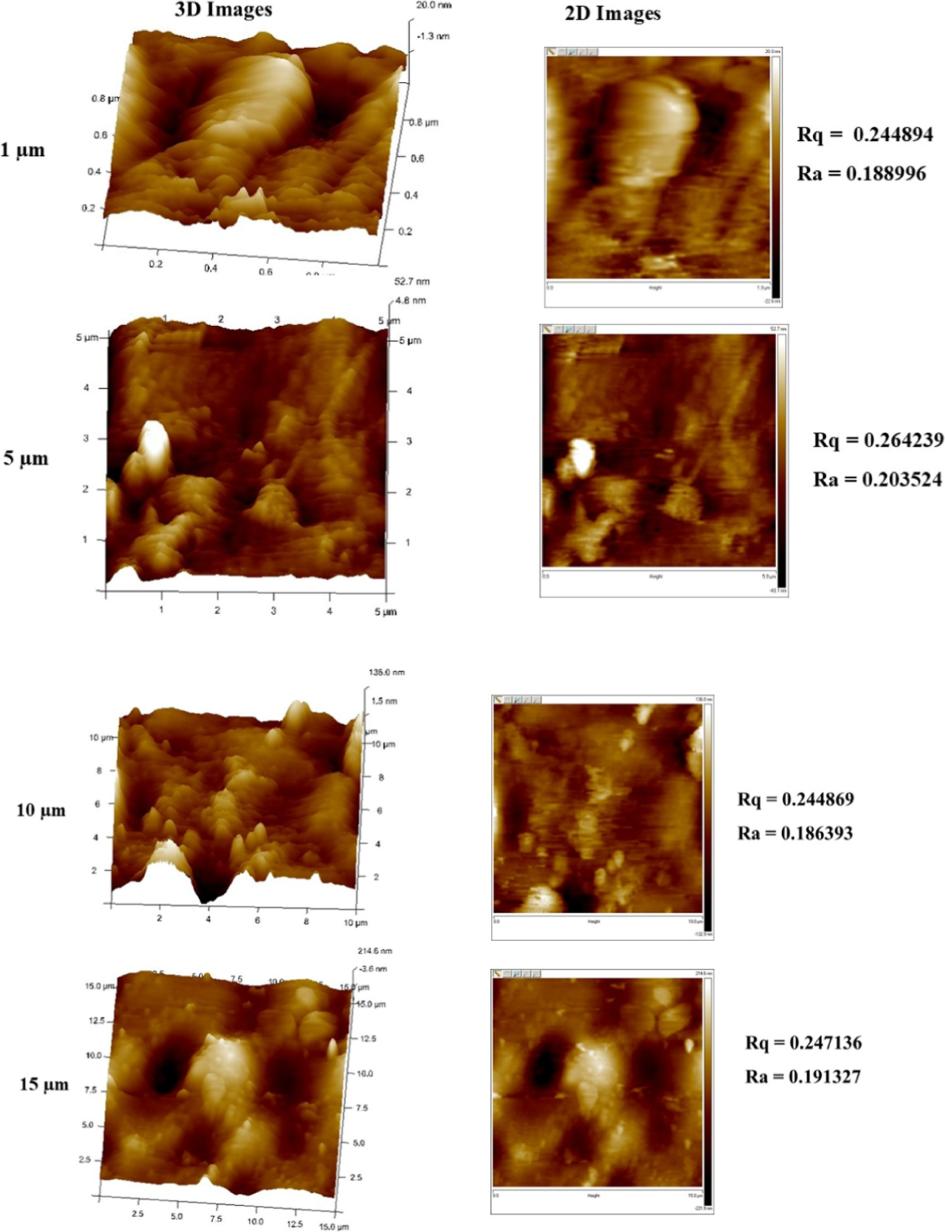
AFM images of CPT film at different magnification areas illustrating the surface topography and roughness characteristics.

The thermal degradation analysis of synthesized chitosan, poly­(vinyl alcohol) (PVA), and tannic acid composite films reveals important insights into their stability and degradation mechanisms. The thermogravimetric analysis (TGA) indicates a moisture desorption process starting at approximately 39.89 °C, which is typical for polymeric materials. As temperature rises, distinct weight loss phases are observed, highlighting the complex thermal behavior of the films. The initial weight loss from 39.89 to 133.60 °C is attributed to moisture evaporation and the decomposition of low molecular weight components within the film.^88^ This phase signifies the onset of thermal instability, where structural integrity begins to diminish. In the intermediate degradation phases, significant weight losses occur between 221.84 and 370.26 °C, indicating more profound structural breakdowns and the release of volatile compounds.^89^ The degradation during this range suggests the breakdown of polymer chains, which is critical for understanding the material’s thermal response. The final degradation phase, from 370.26 to 463.52 °C, shows that the film maintains considerable thermal stability before substantial degradation occurs.^32^ This stability is advantageous for applications requiring materials that can withstand high temperatures. In this study, established equations from thermogravimetric analysis (TGA) were employed to evaluate mass loss during the transition phases, thereby enhancing the understanding of thermal degradation kinetics. This research not only clarifies the thermal stability of the CPT film but also elucidates the degradation mechanisms essential for optimizing its performance in diverse applications. The thermal degradation profiles of the CPT film exhibit a complex response to temperature variations, characterized by distinct phases of weight loss associated with moisture evaporation and polymer degradation. A comprehensive understanding of this behavior is critical for developing materials capable of maintaining functionality under thermal stress conditions. The mass loss for the transitions (Table 2) was calculated using the equation given below

$$rate\;of\;mass\;loss\;\left(mL\right)=\left[\frac{mA1-mB1}{mA1}\right]\times 100$$

**Table 2:** Thermal Properties of Chitosan, PVA, and CPT Film Detailing Key Parameters Such as Weight Loss Transitions and Rates of Mass Loss During Thermal Degradation

film type	weight loss transition (°C)	rate of mass loss %
chitosan film	29.04–148.05	15.95
	148.05–350.34	16.62
PVA film	30.59–148.06	10.61
	239.19–358.25	69.07
	432.14–464.86	4.52
CPT film	39.89–133.60	7.15
	221.84–311.67	36.25
	370.26–463.5	33.85

### 4.9 Atomic Force Microscopy

The analysis of the surface roughness of the CPT film using atomic force microscopy (AFM) reveals significant insights across multiple scales, highlighting the film’s suitability for advanced applications (Figure 9). At a 1 μm scale, the AFM measurements indicate a root mean square (*R*_q_) value of 0.244894 nm and an arithmetic average (*R*_a_) of 0.188996 nm. These values suggest a relatively smooth surface with minimal deviations, which is crucial for applications requiring high precision, such as in electronic and optical devices. When examining the film at a 5 μm scale, the roughness parameters show an increase, with *R*_q_ at 0.264239 nm and *R*_a_ at 0.203524 nm. This slight increase in roughness may reflect variations in surface texture that can influence performance in specific applications, such as coatings where uniformity is essential for adhesion and functionality. At the 10 μm scale, the film displays *R*_q_ values of 0.244869 nm and *R*_a_ of 0.186393 nm, indicating a return to smoother characteristics similar to those observed at 1 μm This consistency across different scales underscores the film’s capability to maintain surface quality, which is vital for thin-film technologies. Finally, at a 15 μm scale, the AFM analysis shows *R*_q_ at 0.247136 nm and *R*_a_ at 0.191327 nm. These measurements further confirm the film’s overall smoothness and uniformity, reinforcing its potential in applications that demand stringent surface specifications. Overall, the AFM characterization across these varying scales demonstrates that the CPT film possesses excellent surface properties with low roughness values, making it suitable for high-precision applications such as thin film technologies and biocompatible coatings. The ability to maintain such low roughness levels across different measurement scales indicates its high quality and expands its application potential in advanced technological fields where surface characteristics are critical for performance and reliability. The significance of smooth surfaces in chitosan, PVA, and tannic acid films for food packaging extends beyond mere aesthetics, it plays a crucial role in enhancing barrier properties and overall functionality. A smooth surface can minimize the permeability of gases and moisture, which is vital for extending the shelf life of food products. This property is particularly important as it helps protect against spoilage and contamination, thereby maintaining food quality.^90,91^ Smooth surfaces facilitate the uniform application of coatings, such as antimicrobial or biodegradable layers, improving the film’s functionality.^92^

### 4.10 Antimicrobial Activity

The efficacy of the synthesized chitosan-based polymer film (CPT) in inhibiting microbial growth was evaluated using the agar cup method against three different microbial strains: the Gram-positive bacterium Staphylococcus aureus MTCC 96, the Gram-negative bacterium Pseudomonas aeruginosa MTCC 1688 and the fungus Aspergillus niger MTCC 282. The CPT film was tested at different concentrations (5, 25, 50, 100, and 250 μg/mL) to determine its ability to inhibit the growth of each microbial strain. The results showed varying levels of antimicrobial efficacy against all the tested microorganisms. For S. aureus, the inhibition zones at different concentrations were as follows (Tables 3 & 4): 0 mm at 5 μg/mL, 9 mm at 25 μg/mL, 10 mm at 50 μg/mL, 12 mm at 100 μg/mL, and 14 mm at 250 μg/mL. However, P. aeruginosa showed a more pronounced inhibitory response with inhibition zones of 0 mm at 5 μg/mL, 10 mm at 25 μg/mL, 11 mm at 50 μg/mL, 13 mm at 100 μg/mL and 16 mm at 250 μg/mL. The fungal strain A. niger showed the lowest susceptibility, with inhibition zones of 0 mm at 5 μg/mL, 7 mm at 25 μg/mL, 8 mm at 50 μg/mL, 10 mm at 100 μg/mL, and 11 mm at 250 μg/mL. The enhanced antimicrobial activity against P. aeruginosa can probably be attributed to the ability of the CPT film to penetrate or destroy the unique outer membrane of Gram-negative bacteria, which is not present in Gram-positive bacteria and fungi. This difference in cell wall structure determines the susceptibility of these microorganisms to antimicrobial agents. The thicker peptidoglycan layer in S. aureus and the different metabolic pathways in A. niger further complicate their susceptibility to the CPT film. These results emphasize the potential of CPT films as effective antimicrobial agents, especially for Gram-negative bacteria. The different responses of the microorganisms tested highlight the importance of preparing antimicrobial materials to target specific pathogens. These results suggest that the mechanisms underlying the antimicrobial action of CPT films need to be further explored, which could improve their effectiveness in biomedical and environmental applications.

**Table 3:** Diameter of the Inhibition Zone Exhibited by CPT Film against S. aureus, P. aeruginosa, and A. niger at Varying Concentrations

zone of Inhibition (mm)															
microbes tested	S. aureus					P. aeruginosa					A. niger				
concentration of polymer sample	5	25	50	100	250	5	25	50	100	250	5	25	50	100	250
polymeric film (CPT)	0	9	10	12	14	0	10	11	13	16	0	7	8	10	11

**Table 4:** Comparative Antimicrobial Efficacy of Standard Antibacterial and Antifungal Drugs against S. aureus, P. aeruginosa, and A. niger at Varying Concentrations, Serving as a Benchmark for Evaluating the Performance of Synthesized Films^a^

antibacterial & antifungal standards															
microbes	S. aureus					P. aeruginosa					A. niger				
concentration of standard drugs	5	25	50	100	250	5	25	50	100	250	5	25	50	100	250
ampicillin	10	13	14	16	18	0	0	0	0	0					
chloramphenicol	12	14	19	20	21	14	17	18	19	21					
ciprofloxacin	17	19	21	22	22	20	23	24	26	27					
norfloxacin	19	22	25	26	28	18	19	21	23	23					
nystatin											18	19	24	29	29

a The inhibition zones demonstrate the activity of standard drugs across different concentrations, providing insights into the relative efficacy of the films.

In this study, the concentration of the CPT film refers to the ratio of the dissolved film material to the volume of the agar solution used during antimicrobial testing. The films were sent to an antimicrobial testing laboratory, where they were dissolved in an appropriate solvent (e.g., DMSO) to prepare stock solutions. These stock solutions were subsequently diluted to achieve varying concentrations which is essential for determining the zone of inhibition.^93^ The antimicrobial activity was assessed using the Agar Cup Method, wherein the diluted solutions were introduced into wells created in a nutrient agar medium (e.g., Mueller Hinton Agar) inoculated with standard bacterial strains (∼10^8^ CFU/mL). After incubation, the diameters of the inhibition zones were measured to evaluate the bactericidal effect of the films.^94^ These measurements provided a quantitative basis for determining the antimicrobial efficacy of the CPT films.

The antibacterial mechanism of chitosan–PVA–tannic acid films is primarily associated with their interaction with bacterial membranes, leading to cell disruption and death. The film releases bioactive components, particularly tannic acid, which chelates metal ions, destabilizes membranes, and generates reactive oxygen species (ROS). This multifaceted approach compromises membrane integrity, resulting in leakage of cellular contents. Additionally, chitosan’s positively charged amino groups enhance this disruption by interacting with the negatively charged bacterial surfaces. Tannic acid disrupts bacterial cell walls by chelating essential metal ions, which is important for maintaining membrane stability. The generation of ROS contributes to oxidative stress, further damaging bacterial cells.^60^ The positively charged amino groups of chitosan facilitate electrostatic interactions with negatively charged bacterial membranes, enhancing membrane permeability and disruption.^95^ The antimicrobial activity has been compared with values reported in the literature for validation (Table S1).

### 4.11 Shelf Life

In a 24 day study, the preservation effect of CPT films for garlic was systematically studied under room temperature conditions (Figure 10).^96–98^ Fresh garlic cloves were encapsulated in the CPT film to prevent access to moisture and aerobic exposure, which are the main catalysts for microbial growth and product deterioration. During the study, encapsulated samples were continuously monitored for color change and microbial spoilage through visual observation. This comprehensive methodological approach enabled a detailed assessment of the ability of the CPT film to preserve the sensory qualities and physiological characteristics of the garlic over time. To provide a baseline for comparison, the study included a control group consisting of unpackaged garlic samples. By comparing the visual changes observed in the film-wrapped garlic compared to the control samples, this study aimed to quantify the effectiveness of the film in inhibiting spoilage processes and extending the shelf life of garlic. A comparative study between the packaged samples and the control group provided significant insights into the preservative capabilities of the CPT film and highlighted its potential as a novel packaging solution for perishable agricultural products. This trial framework provides meaningful data on the ability of CPT film to extend the shelf life of garlic and potentially other similar agricultural products, ultimately reducing food waste and improving the efficiency of the food supply chain.^37,99^

**Figure 10:**
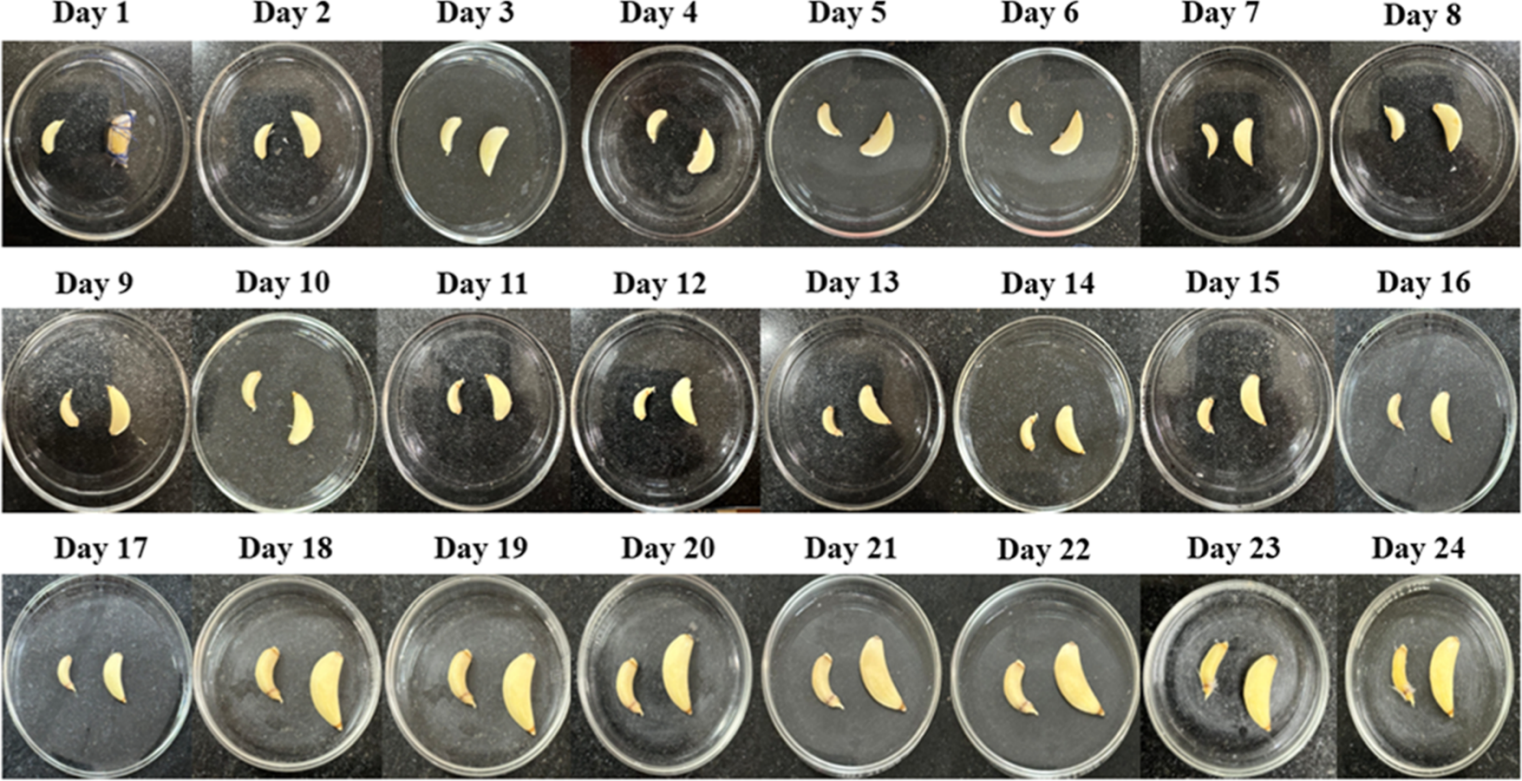
Optical image of the CPT film demonstrating its transparency and suitability for garlic preservation.

In this study, we evaluated the qualitative characteristics of garlic, focusing on color changes and spoilage, through daily visual observation during storage.^100–108^ Garlic packed in film was monitored for visible signs of deterioration, including discoloration, texture changes, and mold growth. This practical method, though lacking adherence to universal instrumental protocols, relied on direct observation to evaluate the film’s effectiveness in preserving garlic quality. The approach offered immediate feedback on the garlic’s condition, enabling timely interventions to address spoilage concerns. A comparison has been made between the findings from our study and those reported in the literature on films prepared using PVA, chitosan, and tannic acid. The table (Table S2) below provides a detailed comparison of the food packaging properties, shelf life, polymeric film prepared, and antimicrobial activity of these films.

## 5 Conclusion

In this study, we developed an innovative, eco-friendly chitosan–PVA–tannic acid (CPT) film using a solvent casting method, marking a significant advancement in food packaging technology. The CPT film demonstrated exceptional potential in extending the shelf life of garlic by protecting it from environmental factors and microbial spoilage. Through SEM and FE-SEM analysis, we confirmed the film’s uniform structure and efficient dispersion of cross-linkers, while the tannic acid-enhanced crystallinity contributed to its superior mechanical properties. With a thickness of 0.03 mm, the film offers excellent flexibility and easy handling. Thermal stability was observed up to 463.52 °C, and AFM analysis showed minimal surface roughness, ensuring product integrity throughout storage. Antimicrobial tests revealed impressive activity against S. aureus, P. aeruginosa, and A. niger, demonstrating the film’s broad-spectrum efficacy. Notably, the CPT film successfully preserved garlic for 24 days, showcasing its potential to reduce spoilage and improve food quality. This multifunctional composite film presents a promising, sustainable alternative to synthetic packaging materials, offering the dual benefit of enhancing food preservation while mitigating environmental pollution. Future research should focus on optimizing its properties for a broader range of perishable goods, positioning CPT films as a key solution in the shift toward more sustainable and efficient food packaging systems.

## Supplementary Material



## Data Availability

The data underlying this study are openly available in [Repository Name] at [Persistent Link to data in Repository, e.g., DOI, Accession Number].

## Notes

The Supporting Information includes Table S1, which compares the inhibition zones of various polymeric films used in food packaging based on their antimicrobial activity, and Table S2, which provides a comparative analysis of polymeric films in terms of composition, functionality, and their effectiveness in enhancing the shelf life of packaged food products (PDF)
