# Supplementary material for: Development and Characterization of Chitosan–PVA–Tannic Acid Film for Extended Shelf Life and Safety of Food Products
Source: ACS Omega. 2025 May 5;10(19):19361–78. doi: 10.1021/acsomega.4c09964 (PMC12096196; doi:10.1021/acsomega.4c09964)
Supplement: Supplementary file 1 [file ao4c09964_si_001.pdf]

Development and Characterization of Chitosan-PVA-Tannic Acid Film for Extended Shelf Life and Safety of Food Products

Sakshi Jasrotia[1], Sonali Gupta[1], Manas Laxman Kudipady[2], Yashoda Malgar Puttaiahgowda[1*]

[1]*Department of Chemistry, Manipal Institute of Technology, Manipal Academy of Higher Education, Manipal, Karnataka, India-576104*

[2]*Department of Information and Communication Technology, Manipal Institute of Technology, Manipal Academy of Higher Education, Manipal, Karnataka, India-576104*

Corresponding author's E-mail: yashoda.mp@manipal.edu

Abstract

The growing demand for sustainable food packaging has led to the development of bio-based and biodegradable materials that minimize the environmental impact of conventional plastics. This study introduces an eco-friendly thin film incorporating tannic acid into a chitosan-polyvinyl alcohol (CPT) matrix to preserve fresh garlic. The film, produced through solvent casting, was thoroughly characterized using several analytical techniques. Fourier-transform infrared spectroscopy (FTIR) and X-ray diffraction (XRD) confirmed strong molecular interactions and increased crystallinity, which enhanced the material's compatibility and structural integrity. Atomic force microscopy (AFM) revealed a surface roughness of 0.167 nm, and thermogravimetric analysis (TGA) demonstrated thermal stability up to 463 °C. The CPT film exhibited notable antimicrobial activity against *Staphylococcus aureus*, *Pseudomonas aeruginosa*, and *Aspergillus niger*. Packaging trials showed that the film effectively extended garlic freshness for 24 days. These findings suggest that the CPT film offers a promising solution for sustainable packaging by combining active food preservation with real-time quality monitoring.

Keywords: Chitosan, Polyvinyl alcohol, Tannic acid, Films, Antimicrobial activity, Food packaging, Shelf life.

The antimicrobial activity has been compared with values reported in the literature for validation **(Table S1).**

Table S1: Comparison of the inhibition zones for polymeric films used in food packaging, including both natural and synthetic polymers, highlighting their antimicrobial activity against *Escherichia coli*, *Staphylococcus aureus*, and *Candida albicans*. The table presents the zones of inhibition for different film compositions, with data from various studies for comparison.

S. No.	Author	Year	Polymeric Film	Antimicrobial Activity	Zones of inhibition	References
1.	Lee et al,	2023	CS-TA0.5/P<br>(Chitosan-Tannic/Phosphate-Buffered Saline)	*Escherichia coli*	4.0 ± 1.2	96
			CS-TA0.5/T<br>(Chitosan-Tannic/Tris Buffer)		3.2 ± 0.8	
			CS-TA1.0/P		10.4 ± 2.0	
			CS-TA1.0/P		10.4 ± 2.0	
			CS	*Staphylococcus. aureus*	3.3 ± 0.4	
			CS-TA0.5/P		4.5±0.8	
			CS-TA0.5/T		3.8±0.2	
			CS-TA1.0/P		13.5±3.2	
			CS-TA1.0/T		13.5±3.2	
2.	Zhang et al,	2023	SA-SF-0.2% tannin	*Staphylococcus aureus*	12.83±0.65	

			(Sodium Alginate-Silk Fibroin Tannin)			97
			SA-SF-0.2% tannin	*Escherichia coli*	9.10±0.74	
			SA-SF-0.5% tannin	*Staphylococcus aureus*	16.24±1.31	
			SA-SF-0.5% tannin	*Escherichia coli*	13.61±0.57	
			Blank SA-SF	*Staphylococcus aureus*	No inhibition	
			Blank SA-SF	*Escherichia coli*	No inhibition	
3.	Xuan et al,	2024	CCTW (Chitosan-Casein-Condensed Tannin-Carnauba Wax)	*Escherichia coli*	6.2±0.37	98
			CCTW	*Staphylococcus aureus*	8.4±0.35	
			CT (Chitosan-Condensed Tannin)		3.4±0.3	
			CC (Chitosan-Casein)		2.6±0.27	
			TACS (Tannin-Alginate-Chitosan-Silk)	*Escherichia coli*	4.6	
			TACS	*Staphylococcus aureus*	4.2	
			CSTA	*Escherichia coli*	2.5	

			(Chitosan-Sodium Tannate)			
			CSTA	*Staphylococcus aureus*	4.4	
			CSNC (Chitosan-Sodium Nanoclay)	*Escherichia coli*	0.43	
			CSNC	*Staphylococcus aureus*	0.53	
4.	Wen et al,	2021	PVA	*Staphylococcus aureus*	No inhibition	18
			PVA	*Escherichia coli*	No inhibition	
			CMCS (Carboxymethyl Chitosan)	*Staphylococcus aureus*	No inhibition	
			CMCS	*Escherichia coli*	No inhibition	
			PVA/15CMCS (Polyvinyl Alcohol - Carboxymethyl Chitosan)	*Staphylococcus aureus*	No inhibition	
			PVA/15CMCS	*Escherichia coli*	No inhibition	
			PVA/15CMCS/2.5CA	*Staphylococcus aureus*	12 mm	
			PVA/15CMCS/5CA	*Staphylococcus aureus*	Varies due to curling	
			PVA/15CMCS/2.5CA	*Escherichia coli*	No inhibition	

			PVA/15CMCS/5CA	*Escherichia coli*	No inhibition<br>No inhibition	
5.	Lingaraj et al,	2023	CSCT-0<br><br>(Cationic Starch/Chitosan Incorporated with Tannic Acid)	*Escherichia coli*	9 ± 1	63
			CSCT-0	*Staphylococcus aureus*	9 ± 1.1	
			CSCT-0	*C. albicans*	8 ± 1.3	
			CSCT-1	*Escherichia coli*	10 ± 1.4	
			CSCT-1	*Staphylococcus aureus*	11 ± 1.0	
			CSCT-1	*C. albicans*	10 ± 1.0	
			CSCT-2	*Escherichia coli*	11.5 ± 1.2	
			CSCT-2	*Staphylococcus aureus*	12 ± 1.5	
			CSCT-2	*C. albicans*	11 ± 1.2	
			CSCT-3	*Escherichia coli*	14 ± 1.6	
			CSCT-3	*Staphylococcus aureus*	17 ± 1.0	
			CSCT-3	*C. albicans*	13 ± 1.6	

A comparison has been made between the findings from our study and those reported in the literature on films prepared using PVA, chitosan, and tannic acid. The table **(Table S2)** below provides a detailed comparison of the food packaging properties, shelf life, polymeric film prepared, and antimicrobial activity of these films.

Table S2: Comparative analysis of polymeric films used in food packaging, highlighting composition, functionality, and potential for sustainable food preservation based on previous research.

S. No.	Author	Year	Polymeric Film	Packaged Food	Shelf Life		Antimicrobial Activity	References
					Film	Days		
1.	Our Work	2024	CS-PVA-TA	Garlic clove	Unpacked	12	*Staphylococcus. aureus, P. aeruginosa, A. niger*	
					CPT	24		
2.	Lee et al	2023	CH/TA composite film	Banana	Unpacked	3	*Escherichia coli Staphylococcus aureus*	96
					Pristine CH	7		
					CH/TA0.5/P	7		
					CH/TA0.5/T	7		
					CH/TA1.0/P	7		
					CH/TA1.0/T	7		
3.	Priyadarshi et al	2018	CH/AKEO composite film	Bread	LDPE Pouch	10	*Bacillus subtilis Escherichia coli*	100
					CH film	10		
					CH/AO.125	10		
					CH/A1.0	10		
4.	Zhang et al	2021	CH/G/TA composite film	Fresh cut apple	CG	10	NA	101
					CH-TA II	10		
					CH-TA III	10		
5.	Tan et al	2015	CH/GFSE composite film	Bread	LDPE	3	NA	102
					CH (0)	5		
					CH (0.5%)	8		
					CH (1%)	9		
					CH (1.5%)	10		
6.	Wen et al	2021	PVA/CMCS composite film		No Packaging	3	*Staphylococcus aureus*	
					PE	5		
					PVA/15 CMCS	5		

				Strawberry	PVA/15 CMCS/2.5 CA	5	*Escherichia coli*	18
7.	Lingaraj et al	2023	CS/CH/TA composite film	Ladyfinger	PE	5	*Staphylococcus aureus Escherichia coli Candida albicans*	63
					CS/CH/TA-0	10		
					CS/CH/TA-0	10		
					Pure CH	3		
					CH-TA & CH-C	7		
					CH-TA & CH-C-TA-W	7		
8.	Xinghai et al	2024	CH/PVA/Cur composite film	Shrimps	CH-C-TA & CH-C-TA-W	5	*Staphylococcus aureus* *Escherichia coli*	103
					CH/PVA-Cur 150	<5		
					CH/PVA-Cur0	3-5		
					CH/PVA-Cur50, CH/PVA-Cur100	5		
9.	Xinghai et al	2024	CH/PVA/Cur composite film	Pork	CH/PVA-Cur0	<5	*Staphylococcus aureus Escherichia coli*	103
10.	Haridevamuthu et al	2024	CH/TCE composite film	Shrimps	Active TCE	Up to 15	*Staphylococcus aureus Escherichia coli*	104
					Neat CH	Up to 12		
					Control	Up to 9		
11.	Yuanjian et al	2024			Unpackaged	< 7		

			PVA/RC composite Film	Strawberry	PE	7	NA	105
					PVA/RC-0.6	7		
					PVA/RC-1.2	7		
12.	Zhang et al	2023	Tannin-based composite film	Fresh cut apple	PE	5	*Staphylococcus aureus* *Escherichia coli*	97
					Control (Without TA)	>5		
					SA/SF-0.3%TA	>7		
					Blank	<5		
13.	Gulden et al	2021	PVA based composite film	Fresh Chicken Fillets	PVOHc	7	*Listeria monocytogenes*	106
					PVOHcLEO	7		
					PVOHcREO	7		
14.	Suganthi et al	2018	Chitosan-based composite film	Carrot inoculated with *E. coli*	Uncovered	<3	*Escherichia coli* *Staphylococcus aureus* *B. subtilis* *P. aeruginosa*	107
					Commercial	3		
					PVA/CA	5		
15.	Mustafa et al	2019	PVA-based composite film	Pasteurized milk	PSB	48	*Methicilliam - Resistant Staphylococcus aureus* *(MRSA)* *Escherichia coli*	108
					PSB5A10PE	48		
					PSB5A20PE	>48		
